# Increased Plasma Brain-Derived Neurotrophic Factor 10.5 h after Intake of Whole Grain Rye-Based Products in Healthy Subjects

**DOI:** 10.3390/nu10081097

**Published:** 2018-08-16

**Authors:** Jonna C. Sandberg, Inger M. E. Björck, Anne C. Nilsson

**Affiliations:** 1 Department of Food Technology, Engineering and Nutrition, Lund University, SE-221 00 Lund, Sweden; 2 Food for Health Science Centre, Lund University, SE-221 00 Lund, Sweden; Inger@innovafood.se

**Keywords:** BDNF, colonic fermentation, cognitive function, dietary fiber, diet intervention, dietary prevention, metabolic regulation, neuronal integrity, rye, type 2 diabetes, whole grain

## Abstract

It has previously been shown in short-term interventions that kernel-based whole grain (WG) rye products have beneficial effects on test markers related to obesity and type 2 diabetes (T2D). T2D increases the risk of several severe health issues, including declined cognitive functions. The protein brain-derived neurotrophic factor (BDNF) is suggested to be a potential biomarker for neuronal integrity. The aim of this study was to investigate the effect on plasma BDNF concentrations, 10.5 h after the intake of WG rye. Healthy young adults were provided late evening meals consisting of WG rye kernel-based bread (RKB) or a white wheat flour-based bread (reference product (WWB)), in a randomized cross-over design. The BDNF concentrations were investigated at fasting in the morning 10.5 h after single evening meals with RKB and WWB, and also after three consecutive evening meals with RKB and WWB, respectively. No difference was observed in the BDNF concentrations depending on the priming setting (*p* > 0.05). The RKB evening meals increased the BDNF concentrations by 27% at fasting (*p* = 0.001), compared to WWB. The increase of BDNF after the RKB indicate that, in addition to anti-diabetic properties, the dietary fiber in WG rye may support neuronal integrity.

## 1. Introduction

The prevalence of type 2 diabetes (T2D) is alarmingly high and is continuously increasing worldwide [1]. T2D is connected to several negative health consequences (e.g., cardiovascular disease [2]). More recently, T2D has also been acknowledged as a risk factor for cognitive impairment and dementia, including Alzheimer’s disease [3]. The prevention of T2D is of major importance, and in this respect, dietary fiber (DF) is widely recommended in a healthy diet aiming at reducing the risk of T2D [4]. In addition, the increased intake of foods rich in DF has been associated with beneficial effects on cognitive functions (e.g., on memory functions [5]). However, the effects of DF intake on cognitive functions have not been widely studied. 

Brain-derived neurotrophic factor (BDNF) is a neurotrophin that is important for neuronal integrity and is mainly located in the areas of the brain that are associated with cognitive control (e.g., memory formation [6]) and energy homeostasis [7]). BDNF is also widely distributed in the gastrointestinal tract [8]. In terms of energy homeostasis, BDNF has been shown to influence appetite suppression [9] and glucose metabolism [10], pointing towards anti-obesogenic and anti-diabetic effects. In addition, reduced levels of BDNF have been detected in patients having, for example, Alzheimer’s disease and depression [6], meanwhile, elevated concentrations of BDNF have been linked to exercise [11]. The ongoing increase in the prevalence of metabolic disorders makes it highly relevant to investigate carbohydrate quality in relation to cardiometabolic parameters, but the acknowledged relationships between the metabolic disorders and cognitive decline make it relevant to investigate the carbohydrate quality, also in relation to cognitive functions. Despite the implications of BDNF as a potential marker of neuronal integrity and brain functions, studies investigating the effects of diet components, for example, whole grain (WG) and DF on BDNF concentrations are scarce.

Previously, we observed an anti-diabetic potential of rye kernel-based meals [12] (e.g., improved glucose tolerance and suppressed appetite variables). The beneficial effects were observed 10.5–13.5 h after intake, and it was proposed that the effects were originated from the colonic fermentation of the DF present in the WG rye. The aim of the present study was to further investigate (in plasma) the potential semi-acute effects of WG rye on the neuronal integrity marker BDNF. The study was a randomized crossover study, including healthy young adults that were provided evening meals consisting of rye kernel-based bread (RKB) or white wheat flour-based bread (WWB), respectively. The evening meals were provided as a single evening meal or as three consecutive evening meals prior to the experimental days. After the last evening meal, the participants fasted until the next morning when the plasma BDNF was measured in the fasting state, 10.5 h after the intake of RKB or WWB.

## 2. Materials and Methods

### 2.1. Ethical Statement 

All of the subjects gave their informed consent for inclusion before they participated in the study. The study was conducted in accordance with the Declaration of Helsinki, and the protocol was approved by the Regional Ethical Review Board in Lund, Sweden (Reference 2013/241) and registered at ClinicalTrials.gov (NCT02093481).

### 2.2. Subjects

The recruitment of test subjects was ongoing during 10 to 31 March 2014, and the experimental work was done by 25 June 2014. Prior to enrollment, 25 subjects were screened and six subjects were excluded, as one or more inclusions criteria were not met. The inclusion criteria were nonsmoker with age between 20–35 years, a body mass index (BMI) between 19–25 kg/m^2^, and with no known metabolic disorders or food allergies. Thus, nineteen healthy subjects (9 men and 10 women), age between 20–33 years old (mean ± standard deviation (SD) = 25.6 ± 3.5 years), and with normal body mass indices (BMI) (mean ± SD: 21.9 ± 1.87 kg/m^2^) completed the study.

### 2.3. Test and Reference Products and Standardized Breakfast

The rye kernel-based test product (bread) (RKB) consisted of 85% whole rye kernels and 15% white wheat flour (cereal dry matter). The reference product was a refined wheat bread (WWB) comprised of 100% white wheat flour (cereal dry matter). The size of the test portions, to be consumed in the evening, was based on 50 g of available starch. The portion sizes for RKB and WWB were 142.5 g and 121.4 g, respectively. Further details regarding the baking procedure of the test and reference products, as well as the standardized breakfast are described elsewhere [12].

### 2.4. Experimental Procedure

The design of the study was a randomized, cross-over trial with approximately one week in-between each test occasions. In total, each test subjects participated at four different experimental days. The test subject consumed each product twice, with different approaches, as follows: (i) as a single evening meal prior to the experimental day, that is, one day consumption (1D); and (ii) as evening meals during three consecutive days (3D) prior to the experimental day. The RKB and WWB were consumed as evening meals at 21:30. After the last evening meal, they were fasting until the following morning (experimental day) when the standardized breakfast was served at the research unit at the Food for Health Science Centre, Lund University. The subjects arrived to the research unit at 07:30 on the experimental days. Blood sampling for the analysis of plasma BDNF was performed at fasting via an antecubital vein using an intravenous cannula (BD Venflon™ Pro Safety Shielded IV Catheter, Becton Dickinson, Franklin Lakes, NJ, USA). Thereafter, a standardized breakfast consisting of white wheat bread and water was served. Further details regarding the composition of the standardized breakfast and additional biomarkers at fasting, and postprandial the breakfast are reported elsewhere (Sandberg et al., 2016 [12]). 

During the three days prior to an experimental day, the subjects were encouraged to standardize their meal pattern, and to avoid food rich in DF and to avoid alcohol. Excessive physical exercise was to be avoided the day prior to the experimental day. Furthermore, no antibiotics or probiotics were permitted two weeks prior and during the experimental period. It was noted that no test subject had consumed antibiotics during the last month. Excel’s Random formula was used to randomly assign the test subject the order of evening meals (RKB or WWB) and length of priming (1D or 3D). Six subjects started with RKB 1D, five subjects started with RKB 3D and four subjects started with WWB 1D respectively WWB 3D. 

### 2.5. Chemical Analyses of Evening Meals and Standardized Breakfast

The evening meal test and reference products, and the standardized breakfast bread were analyzed with respect to the total starch, resistant starch (RS), and non-starch polysaccharides (NSP) (total soluble and total insoluble DF, arabinoxylans, and fructans), see Table 1. The available starch content was calculated by subtracting the RS from the total starch. Information regarding the methods used to determine the total starch, RS, and total DF is provided elsewhere [12]. To analyze the fructo-oligosaccharides and inulin (from here on referred to as fructans) in the test products, the enzymatic/spectrophotometric AOAC method 999.03 was performed, in duplicate, using the enzyme assay kit K-FRUC (Megazyme, Bray, Ireland). The analysis included treatment with α-galactosidase to avoid the possible interference of raffinose-series oligosaccharides [13]. The Uppsala method was used to determine the arabinoxylans content and was done by analyzing the monomeric composition in the isolated DF residue from the test products [14]. The calculation of the amount arabinoxylans was based on the xylose, galactose, and arabinose composition, and the calculations were corrected for the amount of arabinogalactan present by assuming ratio 0.69 between arabinose and galactose [15].

### 2.6. Determination of Plasma BDNF

Venous blood samples intended for BDNF analysis were collected in K_2_EDTA tubes at fasting at all four of the visits. The tubes were centrifuged at 1000× *g* for 10 min (4 °C) within 10 min from being collected, and thereafter the plasma was separated and stored at −80 °C until analysis. The plasma BDNF was determined with an enzyme immunoassay kit (CYT306 (ChemiKine) BDNF Sandwich ELISA kit, Merck Millipore, Billerica, MA, USA).

### 2.7. Calculations and Statistical Methods

Data are expressed as means ± SEM. GraphPad Prism (version 6, GraphPad Software, San Diego, CA, USA) was used for graph plotting. ANOVA (general linear model) was used to assess the significant differences in the BDNF concentrations depending on the different test meals. The data was analyzed in MINITAB Statistical Software (release 17; Minitab, Minitab Inc, State College, PA, USA) as a 2 × 2 factorial design. The test products (RKB and WWB) and the length of priming (1D and 3D) were used as independent main variables, and main effects as well as the interactions were evaluated. Test subjects were included as a random factor in the statistical model. The significance level was set at *p*-values < 0.05. If the significant main effects of the test product were detected, and no significant time × treatment interactions were observed, the test products are referred to as ‘RKB’ (i.e., mean value of RKB 1D and RKB 3D) and ‘WWB’ (i.e., mean value of WWB 1D and WWB 3D), respectively. Similarly, the significant main effects of the days are referred to as ‘1D’ (i.e., mean value of RKB 1D and WWB 1D) and ‘3D’ (i.e., mean value of RKB 3D and WWB 3D), respectively. Consequently, the test products were only distinguished as RKB 1D and WWB 1D, respective RKB 3D and WWB 3D if significant interactions (length of priming × treatment) were detected. If residuals were unevenly distributed (tested with Anderson–Darling and considered unevenly distributed when *p* < 0.05), a Box Cox transformation was performed on the data prior to the ANOVA analysis. One subject was excluded from the statistical analysis including single evening meals (1D) (WWB 1D and RKB 1D, *n* = 18), because of an upper respiratory tract infection during one of the 1D visits, which was discovered after the completed experimental period. The primary outcome measure was change in blood glucose incremental area after the standardized breakfast and number of participants required for the study, determined in MINITAB, using previous blood glucose incremental area results from a study including barley kernel evening meals [16]. Consequently, 13 participants were required when assuming a difference of 70 mmol·min/L between after consuming the WWB or RKB the previous evening, and a SD of 82 mmol·min/L, with α = 0.05 and 1-β = 0.8. However, because of a lack of information regarding semi-acute overnight studies including rye, we increased the number of participants to 19. 

## 3. Results

**Figure 1:**
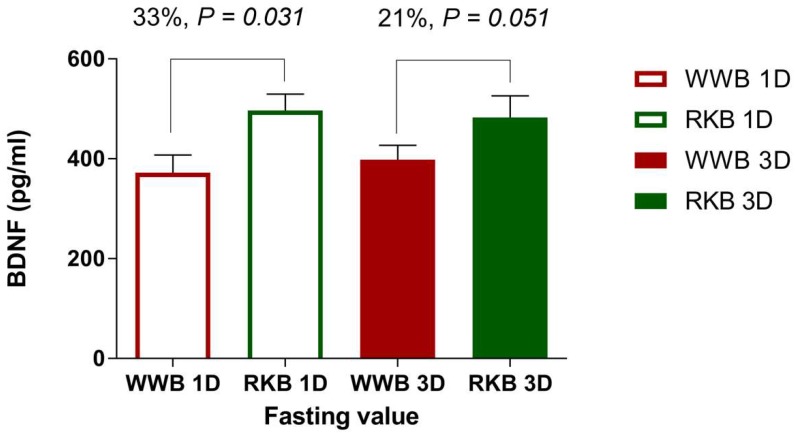
Brain-derived neurotrophic factor (BDNF) concentrations at fasting, 10.5 h post intake of rye kernel-based bread (RKB) or white wheat flour-based bread (WWB) evening meals the mean concentrations of the fasting BDNF in plasma 10.5 h post ingestion of one (1D) or three consecutive (3D) evening meals consisting of rye kernel-based bread (RKB) or white wheat flour-based bread (WWB), respectively. Values are means ± SEM.

The results showed a main effect of the test product on the BDNF concentrations at fasting (*p* = 0.001), revealing significantly increased BDNF concentrations by 27% at fasting 10.5 h after the intake of the RKB compared to the intake of WWB (Figure 1 and Table 2). No main effects on the BDNF of length of priming (1D or 3D), or interactions (length of priming × treatment) were observed. Thus, the results demonstrated that the rye evening meal increased the BDNF concentrations independently of the length of priming.

## 4. Discussion

In the present study, we observed a substantial increase (27%) in the plasma BDNF concentrations 10.5 h after the intake of a rye kernel-based bread, compared to an intake of a white wheat flour-based bread. To the best of our knowledge, this is the first randomized controlled study to report such effects of WG rye or other cereals in healthy adults. The increase in BDNF in this time perspective was of a similar magnitude irrespective of whether the WG rye was ingested as a single evening meal or as three consecutive evening meals. This finding can be added to the other health aspects seen of the rye kernel product on cardiometabolic test variables in the same time perspectives [12]. Consequently, concomitantly with the increased BDNF concentration as observed in the present study, we have previously observed benefits on glucose tolerance and appetite regulation of WG rye in healthy subjects, independent of whether the WG rye was consumed as a single evening meal or as three consecutive evening meals [12].

A decreased concentration of plasma BDNF has been associated with impaired glucose metabolism and T2D [17]. It has been suggested that one possible mechanism behind the promotion of health benefits on glucose tolerance and appetite regulation of WG rye is connected to the gut fermentation of the DF, and production of short chain fatty acids (SCFA) by the gut microbiota [12]. Thus, an increase in plasma butyrate by 30% (*p* < 0.01), and an increase of both plasma acetate (*p* < 0.05) and plasma propionate (*p* = 0.050) by 10% were observed at fasting after the intake of RKB the previous evening, compared to the WWB evening meals [12]. Interestingly, it has been shown that administration of sodium butyrate may upregulate the BDNF levels in the frontal cortex [18]. In terms of the gut microbiota, previous observations have revealed that the BDNF concentrations are lower in germ-free mice compared to controls, and it was concluded that the gut microbiota plays a role in increasing the brain BDNF levels [19]. In addition, another animal study showed that prebiotic feeding (fructo-oligosaccharides and galacto-oligosaccharides) increased the expression of BDNF and peptide YY (PYY), indicating involvement of gut microbiota and gut hormones in rats [20]. However, no correlations between SCFA, gut hormones, and BDNF could be confirmed in this study. In the present study, the results indicate potential semi-acute (10.5 h) effects of WG rye on the neuronal integrity marker BDNF. Further studies are however needed to support this finding. In addition, longer-term interventions have to be executed to determine the lasting impact of WG intake on BDNF concentrations. The present study had limitations. BDNF is associated with brain functions, or example, memory formation. However, cognitive performance tests were not possible to include in the present study, which is an evident constraint. Thus, both shorter- and longer-term effects of WG rye on BDNF and cognitive performance should be investigated in future studies.

## 5. Conclusions

The increase in the BDNF levels after the WG rye in the present study indicate the beneficial effects of WG rye on neuronal integrity and cognitive functions, possibly as a consequence of the gut fermentation of fermentable components in rye and the production of fermentation metabolites, such as SCFA. Consequently, in addition to the previously described anti-diabetic potential of rye, WG rye might have implications for preventing cognitive decline associated with metabolic imbalance. Further investigations are needed to confirm such relationships.

## Figures and Tables

**Table 1:** Carbohydrate composition and portion size of the evening meals RKB and WWB, respectively ^1^

Product	Portion Size	Starch			NSP		Total DF	DF Components	
		**Total**	**Available**	**RS**	**Insoluble**	**Soluble**		**AX**	**Fructans**
	% dry matter								
RKB	-	66.6	61.8	4.8	12.3	3.8	20.9	6.1	4.0
WWB	-	80.5	78.5	2.0	2.6	2.0	6.6	0.5	0.5
	g/day								
RKB	143	53.9	50.0	3.9	9.0	2.8	15.7	5.0	3.2
WWB	121	51.3	50.0	1.3	1.5	1.1	3.9	0.3	0.3

^1^ Data are presented as means. Available starch is calculated as the difference between total starch and resistant starch (RS). Further details regarding the analysis method of the total starch, RS, and soluble and insoluble NSP are presented elsewhere [12]. Analysis of the fructans was performed in duplicates using the enzymatic/spectrophotometric AOAC (Association of Official Agricultural Chemists) method 999.03 [13]. The arabinoxylans (AX) content was analyzed in duplicates using the Uppsala method [14] and was corrected for the amount of arabinogalactan present by assuming a 0.69 ratio between the arabinose and galactose [15]. Total DF include RS and NSP (insoluble and soluble). AX—arabinoxylans; DF—dietary fiber; NSP—non-starch polysaccharides; RKB—rye kernel-based bread; RS—resistant starch; WWB—white wheat flour-based reference bread.

**Table 2:** Fasting plasma brain-derived neurotrophic factor (BDNF) concentrations, 10.5 h following the RKB and WWB evening meals ^1.^

Test Variable	WWB		RKB		%Δ ^2^
	Mean	SEM	Mean	SEM	
BDNF, fasting (pg/mL) ^3^	385.5	22.7	489.6	27.2	27 ***

^1^ Data are presented as means (± SEM) that include data from two test occasions for each product, i.e., ‘RKB’ include RKB consumed as a single evening meal (one day; 1D) and as three consecutive evening meals (three days; 3D), and ‘WWB’ include data from WWB 1D and WWB 3D. ^2^ The percentage change is calculated as the difference from the WWB to RKB. ^3^ *n*_1D_ = 18, *n*_3D_ = 19. *** Different from WWB *p* = 0.001. BDNF, brain-derived neurotrophic factor; RKB, rye kernel-based bread; WWB, white wheat flour-based bread.
